# Chemical Analysis, Qualitative and Quantitative

**Published:** 1849-11

**Authors:** 


### ARTICLE VIII.

*Chemical Analysis, Qualitative and Quantitative.*—By HENRY M. NOAD, Lecturer on chemistry at St. George's Hospital; Author of "Lectures on Electricity," "Lectures on Chemistry," etc., etc. With numerous additions by CAMPBELL MORFIT, Practical and Analytic Chemist; Author of "Chemical aud Pharmaceutic Manipulations," and co-Editor of the "Encyclopædia of Chemistry." With Illustrations. Philadelphia: Lindsay & Blakiston. 1849. pp. 572. 8 vo. (From the Publishers. For sale by S. C. Griggs & Co., Chicago.)

By *Qualitative* Analysis is meant the demonstration of the substances entering into the composition of a compound body:

and by *Quantitative* Analysis, the amount of each ingredient contained in the analysed substance. To the practical chemist a knowledge of Analysis is indispensable; and we have seen no work which so fully treats of the subject as the one before us. Works on chemistry have greatly multiplied within the last few years; but we do not recollect to have seen any one treating of this particular department of the science. We therefore consider this volume as a valuable addition to our chemical literature. In these days of adulterated drugs, honest apothecaries and physicians who dispense their own medicines need to be on the alert against impositions.—To protect themselves from the arts of fraudulent dealers, requires them to possess a knowlege of Analytic Chemistry; and we know of no work so well calculated to afford them the requisite instruction, as the one before us. The toxicological properties of chemicals are fully treated of, together with their antidotes and modes of detection. In this department the latest discoveries are noted down, together with the most approved modes of testing. We recommend this volume to every intelligent physician, as one that will be of service to him, and from the perusal of which he will derive much pleasure. M.

